# Different Swine Production Systems Can Shape Slurry Resistome at Mechanism and Class Levels Based on Swine Manure Evaluation

**DOI:** 10.3389/fcimb.2022.879656

**Published:** 2022-07-04

**Authors:** Lucas Cafferati Beltrame, Caetana Paes Zamparette, Clarissa Feltrin, Caroline Ribeiro da Cunha, Elisa Pires Coltro, Gabriel Saldanha da Silva Athayde, Vilmar Benetti Filho, Deisi Cristina Tápparo, Jamir Monteiro, Jalusa Deon Kich, Jussara Kasuko Palmeiro, Glauber Wagner, Gislaine Fongaro, Carlos Rodrigo Zárate-Bladés, Thaís Cristine Marques Sincero

**Affiliations:** ^1^ Laboratory of Immunoregulation, iREG, Department of Microbiology, Immunology, and Parasitology, Federal University of Santa Catarina, Florianópolis, Brazil; ^2^ Laboratory of Applied Molecular Microbiology, MIMA, Department of Clinical Analysis, Federal University of Santa Catarina, Florianópolis, Brazil; ^3^ Laboratory of Bioinformatics, Department of Microbiology, Immunology, and Parasitology, Federal University of Santa Catarina, Florianópolis, Brazil; ^4^ Embrapa Suínos e Aves, Concórdia, Brazil; ^5^ Faculty of Veterinary Medicine, University of Southern Santa Catarina, Tubarão, Brazil; ^6^ Laboratory of Applied Virology, LVA, Department of Microbiology, Immunology, and Parasitology, Federal University of Santa Catarina, Florianópolis, Brazil

**Keywords:** antimicrobial resistance (AMR), MinION nanopore device^®^, animal husbandry, swine manure (SM), metagenomics, NGS—next-generation sequencing, Pigs (*Sus domesticus*), One Health (OH)

## Abstract

Antimicrobial resistance is a major threat to public health. Antimicrobial use in animal husbandry is a major concern since it can favor an increase in antimicrobial resistance among farms. Herein, we aim to better understand and characterize the main resistome profiles in microbial communities found in pig farms. Sampling of swine manure was performed in two different timepoints (October 2019 and January 2020) in each of the 14 different swine farms, located in the mesoregion of Western Santa Catarina state in Brazil, a pole of swine product production of worldwide importance. Samples were divided into three groups: farms with the opened regimen and no usage of antimicrobials (F1; n = 10), farms with the closed regimen and usage of antimicrobials (F2; n = 16), and farms with the closed regimen and no usage of antimicrobials (F3; n = 2). The metagenomic evaluation was performed to obtain and identify genetic elements related to antimicrobial resistance using nanopore sequencing. We used ResistoXplorer software to perform composition, alpha and beta diversity, and clustering analysis. In addition, PCR reactions were performed to confirm the presence or absence of seven different beta-lactamase family genes and five phosphoethanolamine transferase gene variants clinically relevant. Our findings based on the identification of resistance genes at the mechanism level showed a prevalence of alteration of the drug target (72.3%) profile, followed by drug inactivation (17.5%) and drug efflux (10.1%). We identified predominantly aminoglycosides (45.3%), tetracyclines (15.9%), and multiclass (11,2%) resistance genes. PCoA analysis indicates differences between F1 and F2 profiles. F2 samples showed increased diversity when compared to the F1 group. In addition, herein we first report the identification of *mcr-4* in a slurry sample (C1F1.1) in Santa Catarina State. In general, our findings reinforce that many factors on the practices of animal husbandry are involved in the resistome profile at the mechanism and class levels. Further studies to better understand microbiome and mobilome aspects of these elements are necessary to elucidate transmission pathways between different bacteria and environments.

## Introduction

Antimicrobial resistance is one of the major problems associated with One Health due to its strong relationship with the different microbiomes found in the most diverse environments (Surette and Wright, 2017). Based on a precautionary principle, the use of antimicrobials in animal husbandry has been progressively banned in some European countries in the last 20 years (Roth et al., 2019). However, some studies report low but consistent levels of resistant species in the environment, including food products. Some investigations even indicate the persistence of some species that show resistance to antimicrobials in the intestinal microbiota even in the absence of selective pressure (Jernberg et al., 2010; Devirgiliis et al., 2011).

Any place where antimicrobials are used is considered a resistance reservoir. Humans and animals as well as their environments where they are inserted such as hospitals and society itself, farms, and places of aquaculture are framed as key players in the spread of antimicrobial resistance (Miłobedzka et al., 2022). Concerns about the use of antimicrobials in animal husbandry extend to its use as a growth promoter, especially when there is a discussion about its routine administration and in sub-doses. The incorrect and uncontrolled use of antimicrobials, in addition to leading to increased resistance, altering metabolic pathways, and impairing the integrity of the human, animal, and environmental microbiota, leads to inefficiency of these molecules (Tyrell et al., 2019; Luu et al., 2021). Some studies of antimicrobial resistance surveillance have already found genes of clinical importance along pig husbandry samples. In addition, the possibility of correlating the resistome found in fecal samples, the phenotypic resistance observed, and the location of the farm has already been described (Mencía-Ares et al, 2020). Xiao et al. (2016) reported that genetics, age, geography, and data can all influence the pig microbiota and, consequently, the resistome. Family farming is known as an eco-friendly and sustainable production system. Additionally, this system is characterized by its lower rates of infections and scarce usage of antimicrobial molecules, which is interesting from an antimicrobial resistance perspective (Mencía-Ares et al, 2020).

The state of Santa Catarina in southern Brazil is a well-known producer of swine products of national and worldwide importance and demonstrates to have, from 2019 until 2021, significant growth over the years. Swine husbandry in Santa Catarina responded annually for about 506,100 tons of national exportations, representing about 1.15 billion U$. In September 2021, Santa Catarina was exporting to about 65 different countries worldwide with a record of 57.700 tons of swine meat (Santa Catarina Government, 2021a; Santa Catarina Government, 2021b; Agricultural Research and Rural Extension Company of Santa Catarina, 2022).

The main objective of this study was to evaluate how different practices and environments related to swine husbandry can influence resistome patterns. We performed our evaluations in farms of swine production located in western Santa Catarina.

## Materials and Methods

### Farm Selection, Sample Collection, and Storage

In this study, we included swine farms located in the most important production area in southern Brazil, specifically in the West of the state of Santa Catarina, that exports pork protein worldwide. In this livestock scenario, a total of three production systems were sampled as follows: a) Group “F1” characterized by the open-regime, free animals, without the use of antimicrobials and production of pork for own consumption (i.e., family farming); b) Group “F2” are confinement farms, which apply an intensive closed system for raising finishing pigs (fattening for commercial purposes), using antimicrobials during the production process. Finally, the “F3” group is represented by experimental farms in a research unit, where an intensive closed regimen is applied, with confinement, but there is no administration of antimicrobials.

In these three different types of farms, swine manure was sampled. F1 samples were collected pre and post dry and wet cleaning of the breeding farm (collection 1; C1—fresh manure, and collection 2; C2 after aerobic treatment in ponds/manure; both in October 2019). Sample collections from groups of farms F2 and F3 were carried out at two different timepoints: October 2019 (C1) and January 2020 (C2), with fresh waste. Operating units, the average number of animals, water source, and antimicrobial use of each farm are listed in **Table 1**.

**Table 1:** Operational data from all sampled farms.

Farm code	Municipality	Operational unit	Mean number of animals	Source of water	Waste treatment	Antimicrobial usage
F1.1	Concórdia	Post weaning	650	Superficial source, chlorination	Anaerobic biodigestion	Do not use antimicrobials
F1.2	Concórdia	Post weaning	650	Artesian well	Anaerobic biodigestion	
F1.3	Concórdia	Post weaning	650	Artesian well	Anaerobic biodigestion	
F1.4	Concórdia	Farrowing	650	Artesian well	Anaerobic biodigestion	
F1.5	Concórdia	Farrowing	650	Artesian well	Anaerobic biodigestion	
F2.1	Concórdia	Farrowing	800	Artesian well	Dunghill—fermentation	Penicillins, Cephalosporin, Tetracycline, Macrolides, Lincosamides, Pleuromutilins, Sulfonamides, Quinolones, Rifampicin
F2.2	Concórdia	Post weaning	850	Artesian well	Biodigestion	
F2.3	Xavantina	Post weaning	600	Artesian well	Biodigestion	
F2.4	Seara	Farrowing	650	Artesian well	Biodigestion	
F2.5	Seara	Post weaning	650	Artesian well	Dunghill—fermentation	
F2.6	São Miguel do Oeste	Finisher	600	Artesian well	Biodigestion	
F2.7	Joaçaba	Finisher	600	Artesian well	Biodigestion	
F2.8	Videira	Complete cycle	10,000	Artesian well	Biodigestion	
F3.1	Capinzal	Complete cycle	25	Superficial source, no chlorination	Dunghill—fermentation	Do not use antimicrobials

For such collections, 25 ml of swine manure was collected and placed in sterile tubes with DNA Shield (Zymo Research, Irvine, California, USA) until total submersion of the sample. Samples were stored at 4°C until processing for metagenomic studies. In total, 14 farms participated in the study, five farms with a family farming system (F1 group); eight with a conventional production system and using antimicrobials (F2 group); and one with a conventional system, but without using antimicrobials (F3 group). The municipalities of origin of the samples were Capinzal, Concórdia, Joaçaba, São Miguel do Oeste, Seara, Videira, and Xavantina (**Figure S1**).

### Library Preparation and Metagenomic Sequencing

All samples were submitted to total DNA extraction using Quick-DNA™ HMW MagBead Kit (D6060; Zymo Research, Irving, USA) according to the manufacturer’s recommendation. We used NanoVue™ Plus (28956058; Biochrom™, Holliston, USA) to obtain quantitative and qualitative data from our DNA samples. Metagenomic libraries were prepared using Rapid Barcoding Kit (SQK-RBK004; Oxford Nanopore Technologies, Oxford, UK) with a DNA input of 400 ng per sample. All the steps were performed following the manufacturer’s recommendations. For each run that was performed during 24 h, we used 12 samples with different barcodes each. All flow cells used were FLO-MIN106D (R9.4.1; Oxford Nanopore Technologies, Oxford, UK) model, and we used a MinION sequencing device (Oxford Nanopore Technologies, Oxford, UK).

### Basecalling and Resistome Construction

We used Guppy (v3.4.5; Oxford Nanopore Technologies, Oxford, UK) software to perform basecalling (**Table S1**). Then, we used the EPI2ME platform (https://epi2me.nanoporetech.com/; Oxford Nanopore Technologies, Oxford, UK) and ARMA (Antibiotic Resistance Mapping Application, Oxford Nanopore Technologies, Oxford, UK) pipeline to obtain gene annotation based on CARD (The Comprehensive Antimicrobial Resistance Database; https://card.mcmaster.ca/; McMaster University’s; Ontario; Canada; last entry in June 2021). Reads with a qscore lower than 7 were excluded. Based on the general mechanism of resistance, we classified all genes in three different categories: alteration of drug target, drug efflux, or drug inactivation. We classified all the identified genes based on the antimicrobial class that confers resistance and the resistance mechanism. Genes that confer resistance to more than one antimicrobial class were classified as “multiclass.” Genes that confer resistance to fusidic acid, antibacterial free fatty acid, aminocoumarin, acridine dye, elfamycin, glycylcycline, isoniazid, mupirocin, nitrofuran, nitroimidazole, nucleoside, pactamycin, polyamin, and rifamycin were herein classified as “others.” All images and statistical analysis were performed using ResistoXplorer (http://www.resistoxplorer.no/; Dhariwal et al., 2021) online software. We assessed alpha and beta diversities to visualize how the different identified genes were related within and between groups, respectively. The heatmaps were generated using the Ward clustering algorithm and Bray–Curtis index as clustering distance. For ordination analysis (beta diversity), we used PCoA (principal coordinate analysis) as the ordination method and the Bray–Curtis index as the distance method.

### Conventional PCR and Multiplex PCR for Identification of Resistance Genes

As a complementary method to detect resistance genes of clinical importance in our samples, we performed different conventional and multiplex PCR reactions. The beta-lactamases *bla*_CTX-M-1_, *bla*_CTX-M-2_, *bla*_CTX-M-9_, *bla*_KPC_, and *bla*_NDM_ genes were identified through conventional PCR reactions (Tartari, 2020). The *bla*_SHV_, *bla*_TEM_, *mcr-1*, *mcr-2*, *mcr-3*, *mcr-4*, and *mcr-5* genes were identified using two different multiplex PCR reactions (Lescat et al., 2018; Dallene et al., 2010). All 10 F1 samples and only the first sampling (C1) from the F2 and F3 groups were submitted to these procedures. All primer sequences and more detailed information are reported in **Table S2**.

The reactions for genes *bla*_CTX-M-1_, *bla*_CTX-M-2_, *bla*_CTX-M-9_, *bla*_KPC_, and *bla*_NDM_ were prepared to a final volume of 10 μl, with 5.0 μl of GoTaq^®^ qPCR Master Mix (A6001; Promega^®^, Madison, USA), 2.0 μl of ultrapure water, 1.0 μl of each primer (1.0 μM), and 50 ng of DNA. The program used in a thermal cycler (Veriti 96-well, Applied Biosystems^®^, Foster City, CA, USA) was initially denatured at 95°C for 5 min, followed by 35 cycles divided into three steps: denaturation at 95°C for 30 s, annealing at 63°C for 30 s, and extension to 72°C for 30 s. Finally, the reactions were subjected to 72°C for 7 min for the final extension of the PCR products.

Reactions for amplifying *bla*_SHV_ and *bla*_TEM_ genes were prepared in a final volume of 10 μl, with 4.2 μl of ultrapure water, 2.0 μl of 5× Green GoTaq^®^ Reaction Buffer, Promega^®^, 0.6 μl of MgCl_2_ (1.5 mM), 1.0 μl of deoxyribonucleotide triphosphates (dNTP; 200 μM), 0.5 μl of each of the primers (1.0 μM), 0.2 μl (1 U) of enzyme (GoTaq^®^ DNA Polymerase, Promega^®^; 5 U/μl), and 50 ng of DNA. The program used in a thermal cycler of amplification was a single cycle at 95°C for 5 min, followed by 35 cycles divided into three steps: denaturation at 95°C for 30 s, annealing at 56°C for 40 s, and extension at 72°C for 1 min). At the end, the reactions were submitted to 72°C for 7 min.

For the mcr family, including *mcr-1*, *mcr-2*, *mcr-3*, *mcr-4*, and *mcr-5*, we performed a multiplex PCR reaction. The final volume established for the reaction was 25 μl, which was composed of 12.5 μl of GoTaq^®^ qPCR Master Mix (Promega^®^), 1.1 μl of each of the 10 primers, 0.5 μl of ultrapure water, and 50 ng of DNA. In a Veriti 96-well thermocycler (Applied Biosystems^®^), the samples were subjected to the following conditions of time and temperature: initial denaturation at 94°C for 15 min, followed by 25 cycles divided into three steps: denaturation at 94°C for 30 s, annealing at 58°C for 1 min and 30 s, and extension at 72°C for 1 min. Finally, the samples underwent a final extension process at 72°C for 1 min.

The fragments related to the genes *bla*_CTX-M-1_, *bla*_CTX-M-2_, *bla*_CTX-M-9_, *bla*_KPC_, and *bla*_NDM_ were loaded onto a 2% agarose gel in TBE buffer (89 mM Tris, 89 mM borate, and 2 mM EDTA) with incorporated ethidium bromide (0.5 μg/ml). Running buffer was used (5× Green GoTaq^®^ Flexi Reaction Buffer; Promega^®^) in a 3:5 ratio (buffer:sample) and commercial molecular size marker 100 bp DNA Ladder (Promega^®^). The potential difference established for the run was 80 V in the first 10 min, followed by an additional 40 min at 100 V. On the other hand, multiplex PCR reactions for *bla*_SHV_ and *bla*_TEM_ were loaded onto a 1% agarose gel while the multiplex PCR reactions for *mcr* genes were loaded onto a 1.5% agarose gel. The other parameters of the run were the same as reported above.

We confirmed the identity of each PCR product tested using Sanger sequencing. We purified amplified PCR products using QIAquick Gel Extraction Kit (QIAGEN^®^). The sequencing was performed on the AB 3500 platform (Applied Biosystems) at the company ACTGene Análises Moleculares (Porto Alegre, Brazil). Reactions were prepared in 5 μl of final volume, consisting of 1 μl of one of the primers (forward or reverse), 30 to 60 ng of DNA, and, if necessary, ultrapure water to make up the volume. The results were visualized using Chromas software (Technelysium; http://technelysium.com.au/wp/chromas/). Analyses were performed using the Phred/Phrap/Consed programs (http://www.phrap.org/phredphrapconsed.html) (Ewing and Green, 1998; Ewing et al., 1998) to verify the quality of the sequencing and assembly of the fragments through the forward and reverse strands. Bases with a score equal to zero were excluded from the analysis. The final sequences were used as a query to build a local database, based on identity, from the gene platform of the NCBI (National Center for Biotechnology Information; https://www.ncbi.nlm.nih.gov/) for validation of the sequence identity. AliView software (version 1.27; Larsson, 2014) was used to visualize the alignments between the database sequences local and the previously generated fragment. With the help of the BLAST tool (Basic Local Alignment Search Tool; https://blast.ncbi.nlm.nih.gov/Blast.cgi), information regarding the identity, coverage, and similarity of the sequenced fragments when compared with the variants found in the database was accessed.

## Results

**Figure 1:**
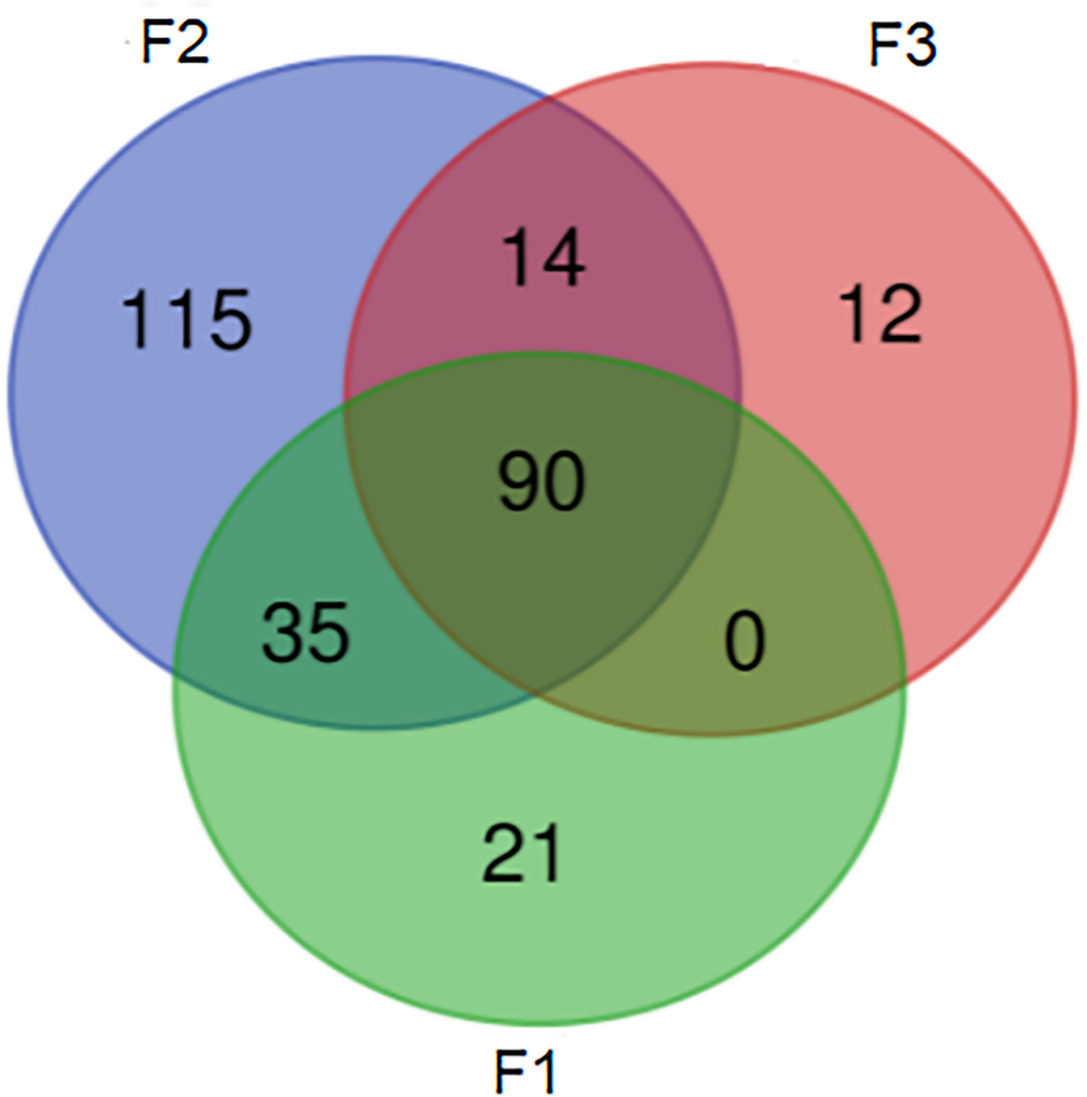
Venn diagram representing common and exclusive genes within groups.

Using nanopore sequencing to access resistome from samples, a total of 287 different genes that confer resistance to different classes of antimicrobials were detected. The quality and yield data of the sequencing are shown in **Table 2**. About 36% of the samples presented a resistome with more than 100 genes, and 80% of these were from conventional system farms (F2 group). The Venn diagram (**Figure 1**) showed that 40% of the genetic elements were found exclusively in F2 group samples. On the other hand, 31% of the genetic elements were found in all the three types of farms.

**Table 2:** Quality control (left) and ARMA reports for all sequenced samples.

Quality Control						ARMA		
Sample	qscore > 7 reads (%)	Reads analyzed	Average sequence length (bp)	Total yield	Average qscore	Reads analyzed	Number of identified genes	Average accuracy (%)
C1F1.1	99.6	3,692	421	1.6 Mb	10.42	102	5	90.7
C2F1.1	99.9	14,885	1,779	26.5 Mb	11.07	1,346	28	85.3
C1F1.2	99.7	2,207	2,776	6.1 Mb	10.79	369	29	89.8
C2F1.2	99.4	1,454	6,794	9.9 Mb	10.64	329	20	81.4
C1F1.3	99.8	944	2,580	2.4 Mb	10.63	129	9	79.9
C2F1.3	99.9	10,371	1,832	19.0 Mb	11.13	1,062	27	81.8
C1F1.4	100.0	20,358	5,121	104.3 Mb	11.66	6,571	115	84.3
C2F1.4	99.3	4,819	441	2.1 Mb	9.88	262	3	79.3
C1F1.5	99.9	18,871	1,350	25.5 Mb	11.26	3,100	38	81.8
C2F1.5	99.6	5,998	2,417	14.5 Mb	10.54	795	13	73.9
C1F2.1	99.8	11,932	3,759	44.9 Mb	11.04	3,295	56	78.2
C2F2.1	99.8	55,570	1,854	103.1 Mb	10.4	3,682	44	76.0
C1F2.2	99.9	50,918	768	39.1 Mb	10.85	3,136	58	82.0
C2F2.2	99.9	690,807	1,000	691.2 Mb	10.66	112,105	107	85.9
C1F2.3	99.9	34,059	936	31.9 Mb	10.91	5,703	65	81.6
C2F2.3	99.9	413,877	2,547	1.1 Gb	10.59	92,528	158	82.7
C1F2.4	99.7	5,140	780	4.0 Mb	10.47	449	10	78.8
C2F2.4	99.9	207,172	492	102.0 Mb	10.5	11,138	108	88.3
C1F2.5	99.9	29,734	522	15.5 Mb	10.85	2,374	116	87.5
C2F2.5	99.9	600,761	850	510.7 Mb	10.66	34,613	101	87.3
C1F2.6	99.9	83,719	727	60.9 Mb	10.97	4,244	71	84.9
C2F2.6	99.9	729,875	1,790	1.3 Gb	10.86	62,080	158	83.1
C1F2.7	99.6	5,248	2,091	11.0 Mb	10.57	554	28	82.2
C2F2.7	99.9	549,989	1,904	1.0 Gb	10.6	40,031	103	81.0
C1F2.8	99.8	8,766	778	6.8 Mb	10.79	508	13	84.3
C2F2.8	99.9	710,049	1,328	943.4 Mb	10.81	74,128	173	84.6
C1F3.1	99.9	59,684	1,002	59.8 Mb	10.98	4,941	59	83.4
C2F3.1	99.9	290,261	627	182.1 Mb	10.55	14,743	114	86.1

bp, base pair.Data Availability Statement.

**Figure 2:**
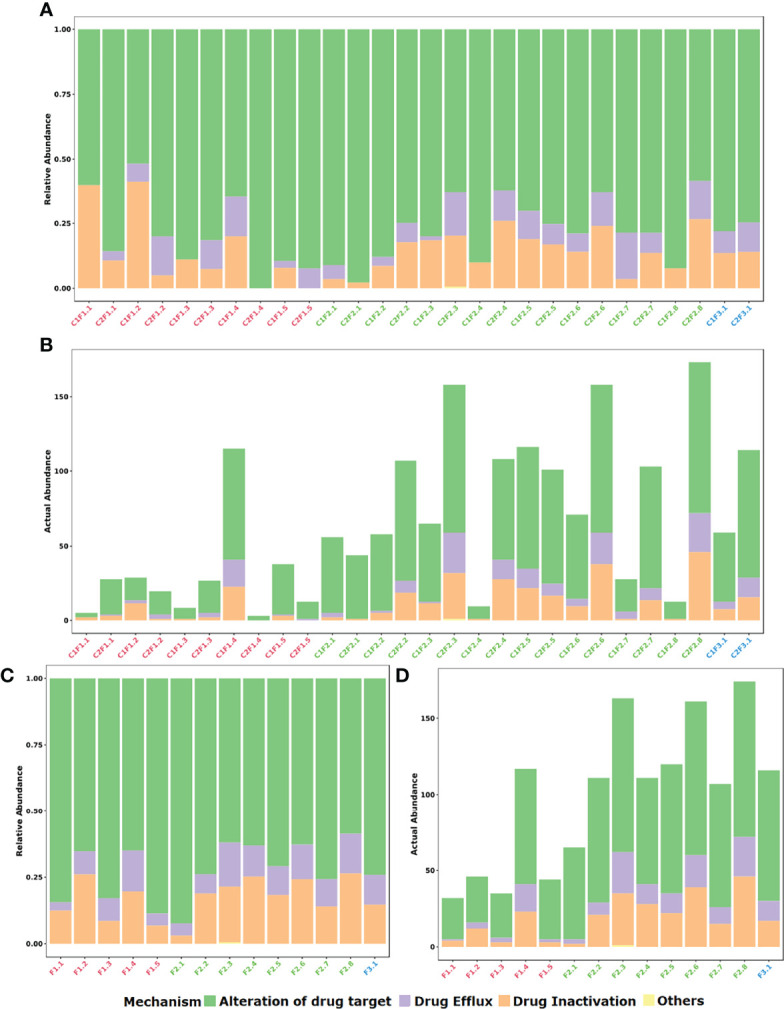
Distribution of identified resistance gene abundance and composition classified by mechanism of action of each gene. **(A)** Stacked bar plot of relative abundance in each sample. **(B)** Stacked bar plot of actual abundance in number of identified genes in each sample. **(C)** Stacked bar plot of relative abundance in each farm considering both samplings. **(D)** Stacked bar plot of actual abundance in number of identified genes in each farm considering both samplings.

Based on the general mechanisms of resistance, a prevalence of alteration of drug target (72.3%) followed by drug inactivation (17.5%) and drug efflux (10.1%) was found (**Figures 2A, B**).

**Figure 3:**
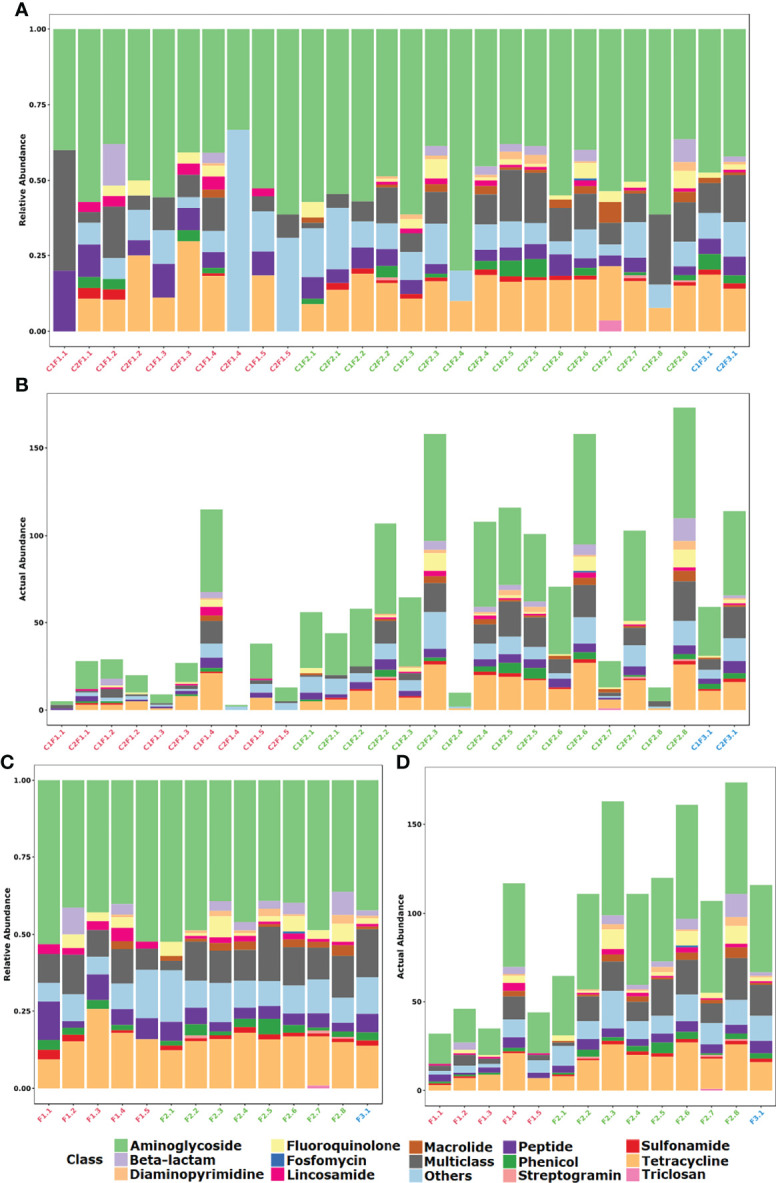
Distribution of identified resistance gene abundance and composition classified by the antimicrobial class that it confers resistance. **(A)** Stacked bar plot of relative abundance in each sample. **(B)** Stacked bar plot of actual abundance in number of identified genes in each sample. **(C)** Stacked bar plot of relative abundance in each farm considering both samplings. **(D)** Stacked bar plot of actual abundance in number of identified genes in each farm considering both samplings.

A majority abundance of genetic elements related to the resistance against aminoglycosides (45.3%), tetracyclines (15.9%), and those characterized as multiclass (11.2%; **Figures 3A, B**) was observed. Sulfonamide resistance based on the presence of *sul* genes were identified in most samples (60.7%) from different farm types. The *erm* gene family was largely found within the samples, mainly between F1 (60%) and F2 (81.2%). Aminoglycoside resistance was represented mainly by mutations in ribosomal gene (53.2%), nucleotidyltransferase (29.9%), phosphotransferase (10.4%), and acetyltransferase (6.5%) enzymes. The OpmH efflux pump that confers resistance to triclosan was found only on a sample from a conventional system farm (C1F2.7).

If the samples of each farm are merged, it is possible to observe that the profile between F1, F2, and F3 groups is essentially similar when the class of antibiotic resistance is considered (**Figures 3C, D**). In contrast, when the mechanism of resistance is compared, the F3 group denotes to be different from groups F1 and F2. Furthermore, at mechanism level there is a prevalence of the alteration of the drug target mechanism among F1 samples in comparison to the other groups (**Figures 2C, D**). On the other hand, it is quite clear the difference between the mean resistome sizes of each group. For groups F1 and F2 that have larger n sizes, we have found 28.7 and 85.5 as the mean numbers of identified genes per sample, respectively.

**Figure 4:**
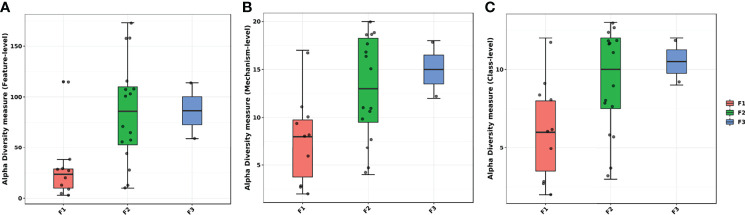
Alpha diversity within groups of the antimicrobial resistance genes classes measured by observed diversity at the **(A)** gene level, **(B)** mechanism of action level, and **(C)** class level. All samples are represented as a dot. Horizontal boxes represent interquartile range and median. Whiskers represent the extreme ranges.

Alpha diversity analyses showed lower observed diversity for F1 when compared to the other groups based on identified genes (**Figure 4A**). It was also possible to notice that the F2 group presented a more scattered diversity level. A class-level and mechanism-level analysis showed similar results in which it was possible to observe that F2 and F3 farms had higher observed diversity levels within the groups, meanwhile F1 presented most of the samples with lower levels of alpha diversity (**Figures 4B, C**).

**Figure 5:**
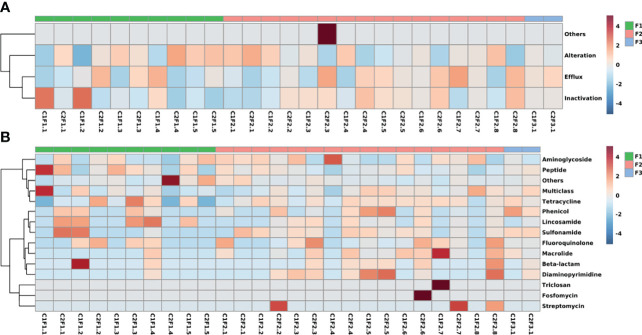
Heatmaps represent the frequency that is classified by **(A)** the mechanisms of action and **(B)** the antimicrobial class that the genes confer resistance. The color spectrum represents the frequency of the class or mechanism as high (red) and low (blue).

From the mechanism perspective (**Figure 5A**), the heatmap shows patterns divided into three different clades. Drug efflux and enzymatic inactivation have more prevalence in samples from F1 and F2 groups as well as drug target alteration mechanisms. At last, samples from all three different groups presented genes from all antibiotic resistance mechanisms.

The class-based heatmap reveals some samples that share similar resistome profiles (**Figure 5B**). Samples were clustered into three different clades. One of them is composed only of two samples (C1F1.1 and C2F1.4) because of their small resistome size which is not suitable to compare with the rest of the samples. Another pattern that can be observed in the heatmap is composed of samples from the three different groups and is characterized by its prevalence of aminoglycoside and peptide resistance. The last group contains mostly F2 samples, which have fluoroquinolone and macrolide resistance genes.

**Figure 6:**
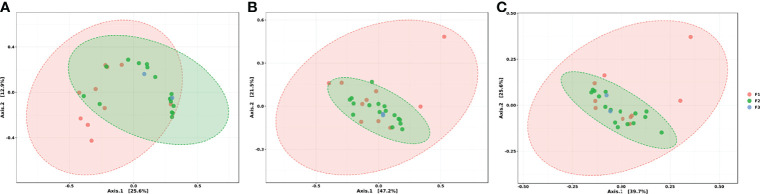
Ordination analysis between groups at **(A)** genes level, **(B)** mechanism of action level and **(C)** class level. PCoA (Principal Coordinates Analysis) using Bray-Curtis Index as distance method. Each sample is represented by a dot. Ellipses were generated for each group, except for the F3 group due to its low n.

By analyzing the beta diversity results, at both the mechanism and class level, we had similar observations (**Figures 6B, C**): the different sample groups had the centroid in common. Conventional swine farms showed very homogenous resistance profiles based on their dispersity. At the same time, those from the F1 group presented greater dispersion in the graph indicating greater heterogeneity. At feature level, we can observe two distinct profiles between F1 and F2 groups, since we have the partial separation of a part of the ellipses (**Figure 6A**).

Next, we aimed to analyze the samples according to their content of genes that have critical clinical relevance, such as those that confer resistance to beta-lactams. Gene families *bla*_OXA_, *bla*_TEM_, and *bla*_CARB_ were the most detected and mainly among samples from the F2 group. The *bla*_OXA-347_ gene was the most frequent gene among those that confer resistance to beta-lactams. It was identified in C1F1.4, C2F2.3, C2F2.4, C1F2.5, C2F2.5, C2F2.6, and C2F2.8 samples. Furthermore, *bla*_TEM-4_ was also detected, an extended spectrum beta-lactamase, in four different samples (C1F2.5, C2F2.5, C2F2.8, and C2F3.1). The class A beta-lactamase gene *cfxA2* that confers resistance to cephamycin was identified in three samples from the F2 group only (C1F2.5, C2F2.5, and C2F2.8).

In the F3 group, despite the small *n* sample, it was possible to observe an increase in the number of genes identified in the resistome in the second collection (C2F3.1; 114 genes) when compared to the first one (C1F3.1; 59 genes). In general, the profile remained similar in terms of both mechanism and class, and even about 50% of the genes identified in this group are present in both samples. The decrease in the relative abundance of genes related to aminoglycosides and tetracyclines and the increase in the relative abundance of genes that confer resistance to more than one class of antimicrobials were evident (**Figures 2**, **3**).

Considering the PCR approach to detect relevant clinical genes, the *bla*_CTX-M-1_ gene was detected in one sample (C1F2.1), as well as *bla*_CTX-M-2_ (C1F1.1). Six different samples were amplified to *bla*_CTX-M-9_ from all the three groups: C1F1.1, C1F1.2, C1F2.2, C1F2.7, C1F2.8, and C1F3.1. No samples presented an amplification for the *bla*_KPC_, *bla*_NDM_, or *bla*_SHV_ genes. Nine different samples were positive to the *bla*_TEM_ gene family: C1F1.2, C1F1.3, C2F1.1, C2F1.2, C2F1.3, C1F2.1, C1F2.4, C1F2.8, and C1F3.1. Finally, only C1F1.1 presented an amplification for the *mcr-4* gene.

Importantly, the presence and identity of all tested genes were confirmed by Sanger sequencing. An identity of about >98% in comparison to our queries were found using all sequences obtained in our local database based on the possible amplified variants from the primers used.

## Discussion

The metagenomic and exploratory analyses performed here were able to provide a comprehensive perspective of the environmental resistome from different pig farm systems based on microbiological and genetic findings. The prevalence of genes that confer resistance against tetracyclines and aminoglycosides and by more than one class of antimicrobials in all the different husbandry regimens was observed. Tetracyclines are among the most widely used antibiotics in the veterinary field to improve physiological performance and to prevent infections (Xu et al., 2020). Some Gram-positive bacteria, such as *Streptomyces aureofaciens*, are well known for being a source of tetracyclines. Its extensive use as a probiotic in animals, especially as an inducer of swine and poultry growth, also allowed the discovery of chlortetracycline (Angelakis, 2017; Otsuka, 2020). Furthermore, most tetracycline resistance genes are found in mobile genetic elements, facilitating its dissemination and transmission between different bacteria (Poirel et al., 2018; He et al., 2019; Sun et al., 2019). Berglund et al. (2020) described environmental microorganisms as the main source of tetracycline resistance elements, and for being an important reservoir.

Resistance to aminoglycosides, often mediated by transferases, are found well dispersed in the most diverse environments. Many studies report the presence of high-abundance resistance genes across clinical and environmental isolates (Khosravi et al., 2017; Galani et al., 2019; Nayme et al., 2019). Furthermore, they are often associated with other genes like ESBLs (extended-spectrum beta-lactamases) or with genetic elements that confer resistance to fluoroquinolones (Krause et al., 2016). The high diversity of genes found in the samples is also related to the extensive use of these antimicrobials in animal husbandry.

The MLS_B_ phenotype, that is, microorganisms that show resistance to macrolides, lincosamides, and streptogramin B, is associated with the *erm* gene family. The results found in this study evidence the widespread dispersion of this family in the animal husbandry environment. Several studies with mobilome approaches of different bacteria species show the strong connection of these genes with the mobile genetic elements such as plasmids and transposons (Yang et al., 2013; Harmer et al., 2015; Xu et al., 2015). Thus, the observed dispersion of the *erm* genes is justified by its relation to mobile elements (Feßler et al., 2018).

Results obtained from distance methods such as PCoA presented some expected data. The fact that conventional regimens (F2) have presented a more homogeneous resistome in comparison to the other systems reaffirms the consequences related to confinement and biosafety practices employed in this type of production system. In the same way, a larger ellipse that we could notice for family farming practices indicates a greater variability found in the resistome from the extensive method that is applied to those animals. Therefore, the type of regimen seems to be directly involved with the construction of the resistome, as the loss of diversity and uniformity in microbial communities are already widely discussed when confinement is practiced (Wang et al., 2021). However, it is known that in family farming environments, there are several interactions between different animal species, which can be another collaborative factor for the greater heterogeneity between samples in this group. Thus, the data suggest that conventional farms have more homogeneous resistomes among themselves, despite the different geographic locations, due to the standardization of practices of production and routine use of antimicrobials. On the other hand, heterogeneity observed among family farming environments may be linked to the subjection of these animals to contamination by the environment (by water, food, etc.), since there is no selective pressure capable of driving the standardization of the profile of resistance and the increase in the number of resistance genes as occurs in conventional production systems. The strict contact between animals and the caretakers is also a key point in microbiological contamination (Conan et al., 2017). This is even more critical within family farming environments, where sanitization protocols are not regularized. Animal-born product consumption is also a concerning practice once indirect contamination can be established (Argudín et al., 2017).

Our findings of identifying genes of clinical importance such as beta-lactamases (e.g., *bla*_OXA_, *bla*_TEM_, *bla*_CARB_, and *cfxA* gene families) or phosphoethanolamine transferases (e.g., *mcr* gene family) are important points of concern within the One Health concept. The detection of the mcr-4 gene by PCR in the C1F1.1 sample can be considered an important finding for the Brazilian scientific literature. The detection of such a gene, more specifically of the *mcr-4.3* variant, was described in 2020 by Martins-Sorenson et al. reporting a clinical isolate of *Acinetobacter baumannii* obtained in 2008 carrying this gene. This isolate would have been identified from the cerebrospinal fluid of a patient with meningitis in Brazil. The identification of this gene in Santa Catarina proves to be a pioneer finding and indicates a great concern about the dissemination of these resistance elements.

On the other hand, the anthropic action within animal husbandry environments is also worrisome in the context of the circulation of resistance genes. The installation of sanitary cesspools near the breeding units, the different sources of water for the animals, and even the health status of the caretakers are important factors in this regard (Regina et al., 2021; Yuan et al., 2022). In this way, urbanization demonstrates to be a critical phenomenon, whether for a controlled environment for animal husbandry such as farms or wildlife (Modesto et al., 2021; Fulham et al., 2022).

Dealing with outbreaks within farms led producers to think about new infection control strategies. Sanitary controls are essential to avoiding further production losses. Control of mortality, serological monitoring, and even sanitization of environments are essential for the eradication of pathogens (de Oliveira et al., 2020). In addition, confinement leads to the compulsory use of antimicrobials to reduce the risk of spreading an alleged infection. Anthropogenic actions negatively influence the health and microbiota of animals, causing less diversity in this ecosystem (Zeineldin et al., 2019). The homogeneity found in conventional farms and, contrastingly not observed in family farming samples, must reflect the practices employed in these two different production systems. There are signs, then, that containment and the use of antimicrobials can reduce the diversity among microbiota within the same group, maintaining a biological standardization. In addition, the determination of the composition of the microbiota of the samples studied is an element that can strongly complement the assessment made in this work. Also, the amount and type of antimicrobials used on farms would be very important data for better understanding the observed resistome profiles.

Bacterial resistance is, in fact, a problem that is difficult to intervene. In this sense, the sanitary systems present on the farms (and even the means that connect urban environments and animal husbandry) must be rethought to the point of avoiding further contamination and circulation of potentially pathogenic organisms. A major challenge for Santa Catarina swine producers and political authorities is to preserve the revenues and worldwide relevance of pork production of this region. Nonetheless, it is needed to perform further analysis to better understand the exact impact that the identification of these genes in the samples can cause over the One Health scenery. A comprehensive microbiome and mobilome assessments could be important to indicate the types of organisms which host different elements and provide some clues about the dynamics in these complex microbial communities.

## Conclusion

In this study, it was possible to observe that the production system can shape the resistome from swine farm slurry due to the different husbandry practices applied in each system. Otherwise, cleaning practices based on scraping and rainwater were also able to diminish the resistome size in F1 samples. In this way, it was clear that what we found predominant, such as resistance against aminoglycosides, tetracyclines, and multiclass, are strictly related, but not only to the use of these antimicrobials for F2 samples. At the same time, F1 and F3 farms are mostly subjected to contamination through drink water and contact with humans. In general, we observed that different practices and environments can lead to different resistome patterns.

## Data Availability Statement

The datasets presented in this study can be found in online repositories. The names of the repository/repositories and accession number(s) can be found below: https://www.ncbi.nlm.nih.gov; PRJNA832246.

## Author Contributions

LB, CZ, CF, CC, JP, and EC performed the sample processing and sequencing experiments. GS, VF, and GW performed the bioinformatic analysis. DT, JM, JK, and GF provided the swine manure samples. LB wrote the first version of this manuscript. GF, CZ-B, and TS conceived the project and worked on the final version of the manuscript. All authors contributed to the article and approved the submitted version.

## Funding

This work was supported, in whole or in part, by research grants from Fundação de Amparo à Pesquisa do Estado de Santa Catarina [FAPESC 2016TR2207]; Conselho Nacional de Desenvolvimento Científico e Tecnológico [CNPq 443808/2018-0]; Coordenação de Aperfeiçoamento de Pessoal de Nível Superior (CAPES); and Bill & Melinda Gates Foundation, Grand Challenges Explorations Brazil – New approaches to characterize the global burden of antimicrobial resistance [grant OPP1193112]. Under the grant conditions of the Bill & Melinda Gates Foundation, a Creative Commons Attribution 4.0 Generic License has already been assigned to the Author Accepted Manuscript version that might arise from this submission.

## Conflict of Interest

The authors declare that the research was conducted in the absence of any commercial or financial relationships that could be construed as a potential conflict of interest

## Publisher’s Note

All claims expressed in this article are solely those of the authors and do not necessarily represent those of their affiliated organizations, or those of the publisher, the editors and the reviewers. Any product that may be evaluated in this article, or claim that may be made by its manufacturer, is not guaranteed or endorsed by the publisher.
