# Supplementary material for: Different Swine Production Systems Can Shape Slurry Resistome at Mechanism and Class Levels Based on Swine Manure Evaluation
Source: Front Cell Infect Microbiol. 2022 Jul 4;12:879656. doi: 10.3389/fcimb.2022.879656 (PMC9289446; doi:10.3389/fcimb.2022.879656)
Supplement: Supplementary file 1 [file DataSheet_1.docx]

Supplementary Material

# Supplementary Figures and Tables

## Supplementary Figures


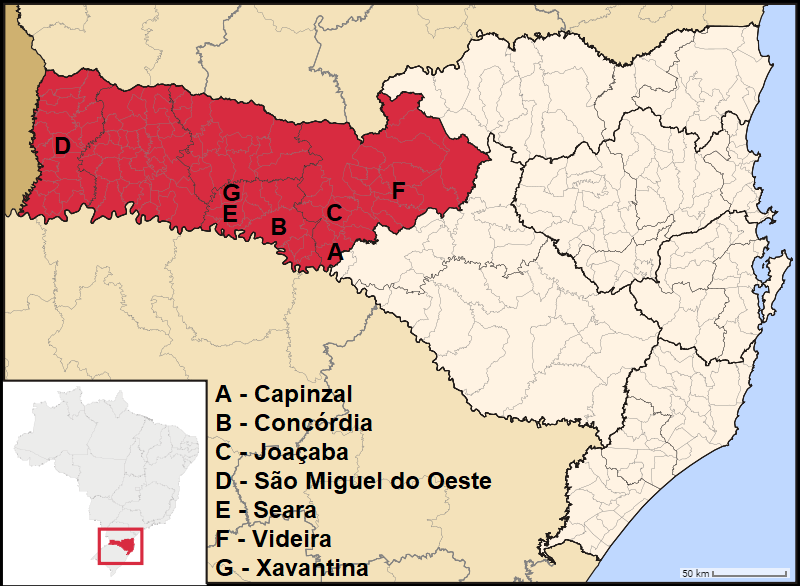


**Supplementary Figure 1.** Map representing municipalities from the Western Santa Catarina (Brazil) where our sampling was performed.

## Supplementary Tables

Supplementary Table 2. Primers used to identify several resistance genes. F: forward; R: reverse; bp: base pair.

| Gene |  | Primer sequence | Fragment lenght | Reference |
| --- | --- | --- | --- | --- |
| *bla*_CTX-M-1_ | F | 5'-GCA AAC TCT GCG GAA TCT GAC G-3' | 182 pb | Tartari, 2020 |
|  | R | 5'-CCG CGA TAT CGT TGG TGG TG-3' |  |  |
| *bla*_CTX-M-2_ | F | 5'-GAT GGC GAC GCT ACC CCT GC-3' | 204 pb | Tartari, 2020 |
|  | R | 5'-GCG GCC GCC ATC ACC TTA CT-3' |  |  |
| *bla*_CTX-M-9_ | F | 5'-AAT CCG ATT GCC GAA AAA CAC G-3' | 135 pb | Tartari, 2020 |
|  | R | 5'-AGC CGT CAC GCC TCC CG-3' |  |  |
| *bla*_KPC_ | F | 5'-GCC GCT GGC TGG CTT TTC TG-3' | 120 pb | Tartari, 2020 |
|  | R | 5'-GCG CCT GAG CCG GTA TCC AT-3' |  |  |
| *bla*_NDM_ | F | 5'-GGT TTC GGG GCA GTC GCT TC-3' | 184 pb | Tartari, 2020 |
|  | R | 5'-CCA TAC CGC CCA TCT TGT CCT G-3' |  |  |
| *bla*_TEM_ | F | 5'-TTC CGT GTC GCC CTT ATT C-3' | 782 pb | Modified from Dallenne *et al*., 2010 |
|  | R | 5'-CCT GAC TCC CCG TCG TGT A-3' |  |  |
| *bla*_SHV_ | F | 5'-GCA AAT TAA ACT AAG CGA AAG CC-3' | 705 pb | Modified from Dallenne *et al*., 2010 |
|  | R | 5'-GTA TCC CGC AGA TAA ATC ACC AC-3' |  |  |
| *mcr-1* | F | 5'-ATG CCA GTT TCT TTC GCG TG-3' | 502 pb | Lescat *et al.*, 2018 |
|  | R | 5'-TCG GCA AAT TGC GCT TTT GGC-3' |  |  |
| *mcr-2* | F | 5'-GAT GGC GGT CTA TCC TGT AT-3' | 379 pb | Lescat *et al.*, 2018 |
|  | R | 5'-AAG GCT GAC ACC CCA TGT CAT-3' |  |  |
| *mcr-3* | F | 5'-ACC AGT AAA TCT GGT GGC GT-3' | 296 pb | Lescat *et al.*, 2018 |
|  | R | 5'-AGG ACA ACC TCG TCA TAG CA-3' |  |  |
| *mcr-4* | F | 5'-TTG CAG ACG CCC ATG GAA TA-3' | 207 pb | Lescat *et al.*, 2018 |
|  | R | 5'-GCC GCA TGA GCT AGT ATC GT-3' |  |  |
| *mcr-5* | F | 5'-GGA CGC GAC TCC CTA ACT TC-3' | 608 pb | Lescat *et al.*, 2018 |
|  | R | 5'-ACA ACC AGT ACG AGA GCA CG-3' |  |  |
